# Virtual herbal garden (*Hayushasutra*): An educational aid for Ayurveda students

**DOI:** 10.1016/j.jaim.2025.101269

**Published:** 2026-01-29

**Authors:** Ramakrishna Allam, B. Kothainayagi, P.A. Sudhir, Vanitha R. Muralikumar, Muthuvel Arumugam, C.R. Rene Robin, S. Yokesh, U. Vishali, S. Fahima

**Affiliations:** a Sri Sairam Ayurveda Medical College and Research Centre, The T.N. Dr. M.G.R Medical University, Chennai, Tamilnadu, India; b Sri Sairam Techno Incubator Foundation, Chennai, Tamilnadu, India; c Sri Sairam Engineering College, Chennai, Tamilnadu, India

**Keywords:** Virtual reality, Medicinal plants, 3D model, Ayurveda, Interactive learning

## Abstract

The AYUSH sector relies mainly on medicinal plants and metals, which form the foundation of traditional healing practices. However, all plants are often inaccessible to many students due to geographic and seasonal limitations. To bridge this gap, Ayurveda Medical College, in collaboration with, Incubation Center and Engineering College, initiated a pilot project to develop a Virtual Herbal Garden a Solution for problem statement given by All India Institute of Ayurveda, under Smart India Hackathon 2024. This digital platform offers an immersive, interactive, and user-friendly learning experience, enabling users to explore medicinal plants in detail. The garden includes 3D models, multimedia resources, and comprehensive information on five medicinal plants: *Cassia fistula*, *Ocimum sanctum*, *Aloe vera*, *Mentha piperita*, and *Azadirachta indica*. The Virtual Herbal Garden is expected to become a valuable educational tool, promoting awareness and understanding of plants used in traditional medicine in the AYUSH sector.

## 1 Introduction

AR (Augmented Reality) and VR (Virtual Reality) are revolutionizing higher education [1], particularly in the medical field, by providing immersive and interactive learning experiences [2]. In general education, AR improves understanding by overlaying digital information onto real-world settings, benefiting fields like engineering, architecture, and science. In medical education, AR enables students to explore complex anatomy by projecting digital models. VR creates fully virtual environments where they can diagnose conditions, and practice surgeries, explore the human body in 3D [3]. These technologies offer hands-on learning in a risk-free setting, enhance deeper engagement, and improve retention, effectively bridging the gap between theoretical knowledge and practical application across various disciplines [4].

The AYUSH sector encompasses to the traditional system of healthcare in India that promotes Ayurveda, Yoga and Naturopathy, Unani, Siddha, Sowa-Rigpa, and Homoeopathy [5]. All these conventional systems of medicine, relies on the therapeutic properties of medicinal plants [6]. Ayurveda students need direct access to these plants to understand their morphology, cultivation methods and medicinal applications. However, physical herbal gardens in educational institutions often face challenges such as limited resources and land constraints, which restrict the diversity of plants that can be grown. The geographical location also plays a role, as plants native to specific regions, like those from the Himalayas, may not flourish in other climates. Furthermore, the seasonality of plants affects their growth, with flowering and fruiting occurring at specific times of the year, limiting year-round access to certain species for study [7,8]. To address these issues, digital solutions like the Virtual Herbal Garden have been developed, providing an innovative approach to studying medicinal plants.

This pilot project, initiated by Ayurveda Medical College, in collaboration with Incubation Center and Engineering College, aims to develop a Virtual Herbal Garden that provides students, and enthusiasts of the AYUSH sector with a comprehensive learning tool as a Solution for problem statement given by All India Institute of Ayurveda, under Smart India Hackathon 2024. By incorporating realistic 3D models, multimedia content, and detailed information, this digital platform allows users to explore medicinal plants from anywhere in the world. This article describes the development process, materials and methods used, and the outcomes of the project, focusing on the educational benefits of integrating technology into Ayurveda education.

## 2 Methodology

**Fig. 1:**
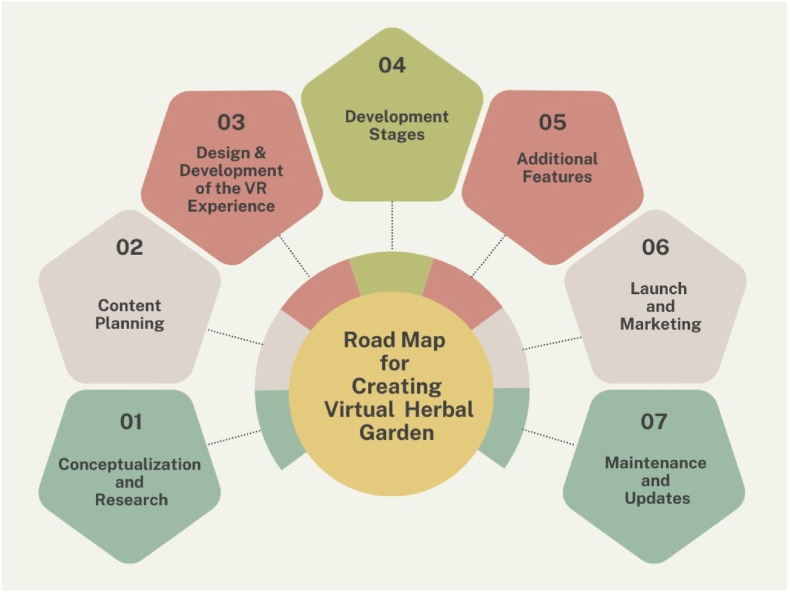
Roadmap for the creation of a virtual herbal garden.

**Fig. 2:**
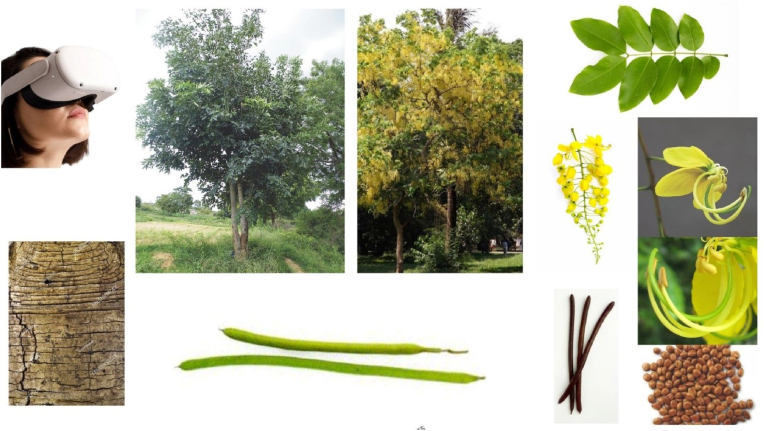
*Cassia fistula,* and its parts.

The development of the Virtual Reality (VR) Herbal Garden for Ayurveda plants followed a structured methodology encompassing seven key stages (Fig. 1) of the AR and VR application development cycle. The first stage, conceptualization and research, involved defining clear objectives such as education, interactive learning, and raising awareness about Ayurvedic herbs. This was followed by an in-depth literature review to gather information on commonly used Ayurvedic plants. Identifying the target audience, including students, practitioners, and researchers, helped tailor the garden's content for maximum educational impact. The second stage, content planning, involved selecting key plant species and creating a comprehensive list of visual elements like plants and their different parts (Fig. 2). Educational modules were integrated, which included audio-visual guides and interactive quizzes to explain each plant's medicinal properties in Ayurveda.

The third and fourth stages, design & development of the VR Experience and Development Stages, focused on building the virtual environment and interactions. 3D models of the plants were designed using software like Blender, and platforms like Oculus and HTC Vive were selected for VR integration. The garden was crafted as an immersive, interactive space where users could walk through, pick plants, and learn about their morphology, phytochemical properties and medicinal uses. Programming interactions through game engines such as Unity ensured smooth functionality. The project also included testing phases to refine user experience and educational content. Additional features like Augmented Reality (AR) integration, multi-language support, and simulation of Ayurveda practices are also planned to further enhance the virtual garden's accessibility and functionality. Regular maintenance and updates were planned to add more plants, keep the content relevant, and update the software compatible with evolving technology.

## 3 Results

### 3.1 Project design and collaboration

The Virtual Herbal Garden project was developed through a multidisciplinary collaboration between Ayurveda Medical College, Incubation Center and Engineering College. The project combined the expertise of Ayurveda faculty with the technical capabilities of the Techno Incubator and Engineering teams. The focus was on creating a user-friendly, interactive platform that simulates the experience of exploring a real-life herbal garden.

### 3.2 Selection of medicinal plants

Five medicinal plants were selected for this pilot project, based on their frequent appearance in UG curriculum, therapeutic diversity, and feasibility for accurate 3D modeling.

• *Cassia fistula* (*Aragwadha*)
• *Ocimum sanctum* (*Tulasi*)
• *Aloe vera* (*Kumari*)
• *Mentha piperita* (*Putiha*)
• *Azadirachta indica* (*Nimba*)

### 3.3 3D model development

**Fig. 3:**
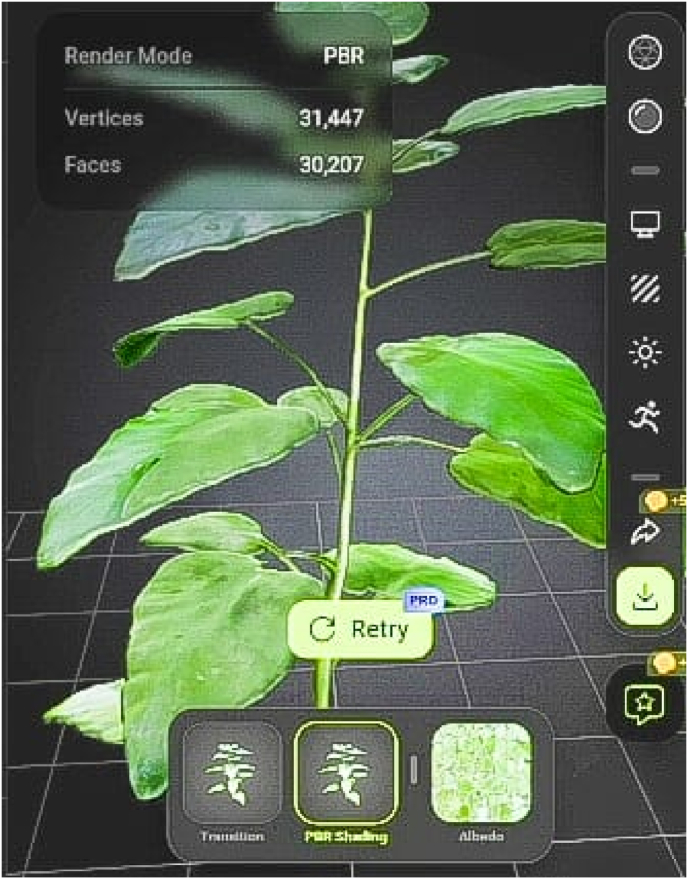
3D model of *Ocimum sanctum*.

**Fig. 4:**
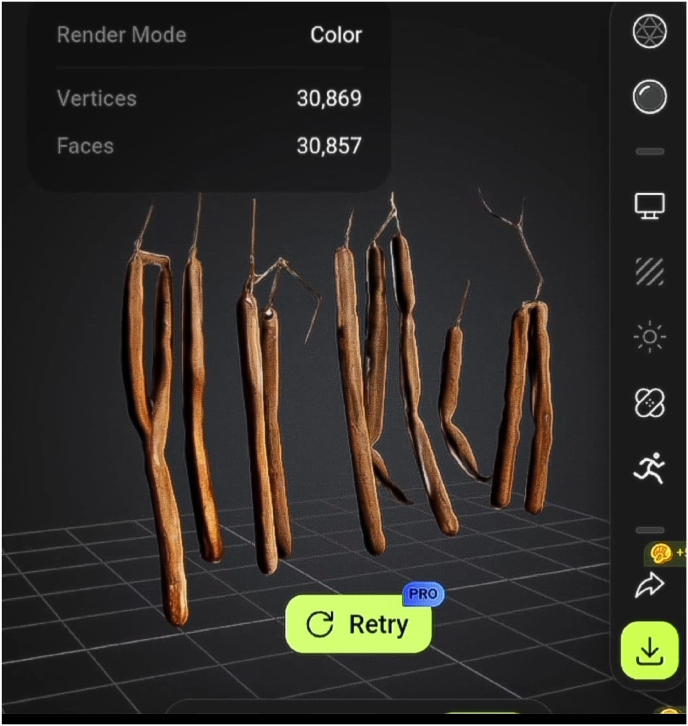
3D model of fruits of *Cassia fistula*.

3D models of the plants were designed using Blender, programmed in Unity 3D, and optimized for Oculus and HTC Vive headsets. The models allow users to rotate, zoom, and explore each plant from different angles, replicating the tactile experience of a physical garden. Each model includes annotations highlighting key morphological features, such as roots, leaves (Fig. 3), flowers, fruits (Fig. 4) and seeds. They serve as interactive teaching aids, mirroring the observational approach followed in physical herbal garden.

### 3.4 Database creation and information integration

A comprehensive database was built for each plant. This information was sourced from Ayurveda Pharmacopoeia of India [9,10], a government-approved text that provides authoritative details on Ayurvedic plants, their pharmacological actions, and formulations and text book of *Dravyaguna vijanana* [11]. These are key resource for aligning traditional medicinal plant knowledge with regulatory and clinical standards, modern botanical books like Trease and Evans' Pharmacognosy [12], which are widely respected textbooks that provide scientific information on medicinal plants morphology and research articles, to make sure the garden is up-to-date with both traditional and modern botanical science [13], research articles to provide recent studies on medicinal plants. These studies help verify the efficacy, safety and pharmacological properties of both conventional wisdom and contemporary scientific knowledge.

The Virtual Herbal Garden would pull from these rich sources to ensure that the information presented balances traditional Ayurvedic knowledge with modern scientific findings, allowing users to learn about:

• Botanical and vernacular names
• Habitat and geographic distribution
• Pharmacognosy of plants
• Properties of Medicinal plants in Ayurveda
• Phytochemical content and pharmacological actions based on modern research
• Methods of cultivation
• Environmental requirements (climate, soil type, water needs)
• Therapeutic uses according to Ayurvedic principles
• Clinical applications with cross-references to modern pharmacology, regulatory guidelines, and dosage forms.

### 3.5 Multimedia integration

To enhance the learning experience, the virtual platform incorporates high-quality images, videos, and audio descriptions for each plant. Video tutorials on plant identification, cultivation methods, and Ayurvedic uses were produced and integrated into the platform. These multimedia resources provide an engaging, multisensory learning experience for users.

### 3.6 Search and filter functionality

An advanced search and filter system was developed to allow users to locate plants based on various criteria, such as medicinal use, geographic region, or therapeutic category (e.g., digestive health, immunity, or skincare). This feature ensures ease of navigation, allowing users to tailor their exploration to specific learning needs.

### 3.7 Virtual tours and user interaction

Virtual tours focusing on specific health themes (e.g., plants for digestive health, immunity, or skin care) were designed to guide users through relevant plants in a thematic, organized manner. Additionally, the platform allows users to bookmark their favorite plants, take notes, and share information on social media, encouraging interaction and collaboration among students and practitioners.

The pilot project successfully created a fully functional Virtual Herbal Garden featuring five medicinal plants. Since this is a pilot project, we have completed the stages of Conceptualization and Research, Content Planning, Design & Development of the VR Experience, Development Stages. We are now concentrating on Pilot Testing and Collecting Feedback, which are crucial for refining the VR experience. This stage is essential for ensuring the product's effectiveness before its launch. The 3D models and multimedia content were well-received by students, who appreciated the visual appeal, ease of use, and depth of information provided. Students reported an enhanced understanding of plant morphology and medicinal properties, particularly benefiting from the ability to interact with the plant models in a virtual environment.

Preliminary user feedback highlighted the value of the search and filter functionality, which enabled them to focus on specific medicinal uses and regions of interest. Additionally, the virtual tours were praised for their structured approach to exploring themes within Ayurveda, offering guided learning experiences that complemented traditional education methods.

## 4 Discussion

The development of the Virtual Herbal Garden (Hayushasutra) represents a significant advancement in Ayurveda education. By overcoming the limitations of physical herbal gardens, such as geographic constraints, seasonal unavailability, and limited institutional access, the platform offers continuous and standardized exposure to medicinal plants for students across various locations. Through the use of 3D models, original standardized images and videos, and multimedia content, the virtual garden creates an engaging and immersive learning experience. This interactive format enhances conceptual understanding and knowledge retention compared to traditional textbook-based approaches.

In this pilot phase, five plants were deliberately chosen based on curriculum relevance, therapeutic diversity, and suitability for accurate 3D modeling to ensure quality and feasibility of development. The design and development stage primarily focused on creating visually realistic and botanically accurate 3D models of plants and their morphological parts (roots, stems, leaves, flowers, fruits, and seeds) were created using Blender. High-resolution textures applied to replicate natural colors and surface features. To ensure botanical accuracy, photographs and videos were captured from the institutional herbal garden under the guidance of Ayurveda faculty, which served as the primary reference material. The models were then optimized for VR by reducing polygon counts without compromising realism, allowing for smooth rendering across different devices. In addition to plant modeling, the surrounding environment, such as pathways, soil textures, water bodies, and interactive signboards, was carefully designed to simulate the immersive experience of walking through a real herbal garden.

Following this, the development stage employed the Unity 3D game engine to integrate plant models into an interactive environment. User interactions such as walking, rotating, zooming, and selecting plants were programmed through C# scripts, while additional features included pop-up panels displaying plant morphology, Ayurvedic properties, phytochemistry, and therapeutic uses. Accessibility was enhanced with bilingual audio narration (English and a regional language) and interactive quizzes linked to plant models for self-assessment. Hardware testing on Oculus Rift and HTC Vive ensured compatibility with major VR platforms, with teleportation-based navigation and hand controllers minimizing motion sickness and simplifying interaction. Usability testing with students and faculty provided valuable feedback, leading to refinements in interface design and the addition of a search-and-filter function for locating plants by therapeutic category.

While the present model demonstrates the feasibility and utility of virtual tools in Ayurvedic herbal education, it offers significant potential for further enhancement. One major area of development is the expansion of the species database to include classical groupings such as *Misraka Gana*, *Mahakashaya*, and other therapeutic categories drawn from authoritative texts like the *Charaka Samhita* and *Sushruta Samhita*. Incorporating regional botanical variants will also reflect the ecological and ethnomedicinal diversity across India, enriching the contextual understanding of students.

Another promising frontier is the integration of olfactory cues. While the current version simulates plant aromas through descriptive analogies, future iterations may incorporate emerging digital scent technologies, such as wearable olfactory emitters or VR-compatible scent cartridges, to facilitate a truly multisensory learning experience. This would align with Ayurveda's pedagogical emphasis on the five sensory modalities (*Pancha Jnanendriyas*), particularly in the context of *Dravya Guna Vigyana*.

Moreover, the platform aims to evolve into a multidisciplinary educational tool by incorporating medicinal plant data and classical references from other AYUSH disciplines, including Siddha and Unani. This integrative approach would broaden the platform's relevance, foster cross-disciplinary learning, and support a more holistic understanding of India's traditional systems of medicine.

To address the challenge of building a comprehensive and scalable digital repository, the project will be executed in a phased manner, starting with the selection of key medicinal plants based on consultations with subject experts. Considering that many plants are distributed across diverse geographical regions and exhibit seasonal variations, the creation of a robust database requires a collaborative and community-driven approach. Partnerships with AYUSH institutions, research centers, forest departments, and botanists will play a crucial role in sourcing authentic plant, thereby facilitating the creation of a national-level standardized repository. All content contributions must adhere to standard image and video documentation guidelines to maintain scientific accuracy, quality, and educational value.

Importantly, the virtual herbal garden is envisioned as a supplementary educational tool that enhances rather than replaces real-world learning. It aims to stimulate student interest and enthusiasm for plant identification, fieldwork, and live herbal garden visits. By integrating traditional Ayurvedic wisdom with modern educational technology, Hayushasutra offers a scalable and innovative model for enriching herbal education in the digital era.

## 5 Conclusion

The Virtual Herbal Garden developed as a pilot project by Ayurveda Medical College, in collaboration with incubation center and Engineering College, provides an innovative and accessible educational tool for the AYUSH students. By offering a digital platform that includes interactive 3D models, multimedia content, and comprehensive plant data, the garden enhances the traditional study of Ayurveda and promotes wider access to knowledge about medicinal plants. As the platform evolves, it has the potential to serve not only as a resource for students and practitioners but also as a public educational tool, promoting awareness and understanding of traditional herbal medicine globally.

This pilot project lays the foundation for further development, with plans to expand the virtual herbal garden to include more plant species, additional interactive features, and advanced AR capabilities. The successful integration of technology into Ayurvedic education through this project demonstrates the potential for digital tools to transform traditional learning practices, ensuring that ancient knowledge is preserved and accessible to future generations in innovative ways.

## Author contributions

ARK: Conception, conceptualizing, original draft preparation, reviewing and editing, BK: Supervision, PS: Supervision, VM: Supervision, MV: Supervision, RR: Supervision YO: Original data curation, draft preparation VS: Analysis, FM: pictures.

## Declaration of generative AI in scientific writing

We hereby declare that no generative AI tools were used in the drafting, writing, or editing of this manuscript. All writing and editing were performed by the authors.

## Funding sources

This article did not receive any funds.

## Conflict of interest

None
